# An Indian female presenting with appendicular diverticulitis: a case report and review of the literature

**DOI:** 10.4076/1757-1626-2-8074

**Published:** 2009-09-09

**Authors:** Sandip K Halder, Imran Khan

**Affiliations:** 1 Department of General Surgery, R G Kar Medical College, Khudiram Bose Sarani, Kolkata, West Bengal, 700004, India

## Abstract

A 29-year-old Indian female patient presented clinically as a case of acute appendicitis. Peroperative finding showed inflamed diverticula of an appendix without perforation. Macroscopically, the rest of the appendix appeared normal. Histopathological examination confirmed appendicular diverticulitis in a noninflamed appendix. The vermiform appendix can rarely be a site of development of diverticula which may be inflamed or noninflamed, with or without appendicitis. Appendicular diverticulosis can present either with chronic abdominal pain or with acute abdominal pain as acute appendicitis. Thay may be completely asymptomatic. It can be associated with various complications resulting increased morbidities and mortalities.

## Introduction

The vermiform appendix can rarely be a site of development of diverticula which may be found inflamed or non-inflamed, with or without appendicitis [1]. Appendicular diveticulitis was first described in 1893 by Kelynac [2]. Although it can be a source of chronic abdominal pain [3], it commonly presents with acute right iliac fossa pain. Occasionally, barium enema can pick up diverticulosis of appendix. Although computed tomogram [4], and ultrasonogram have diagnosed appendicular diverticulitis, it is commonly diagnosed peroperatively. As it can be associated with various life threatening complications like perforation, peritonitis, abscess, pseudomyxoma peritonei, the treatment should be appendicectomy.

## Case presentation

**Figure 1:**
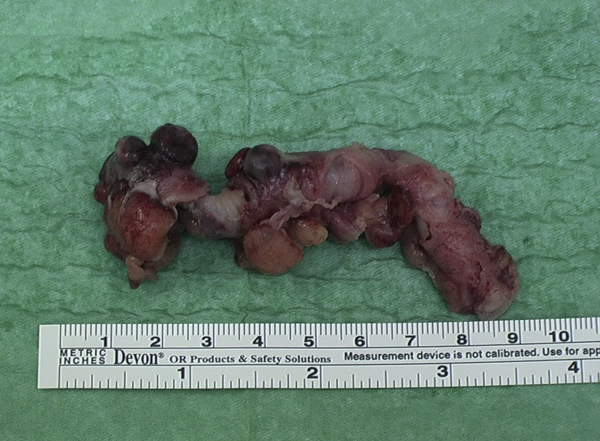
**Specimen of normal appendix with inflamed diverticula**.

A 29-year-old Indian female patient presented with a two day history of progressively worsening localized right iliac fossa pain of acute onset, fever, nausea and anorexia. Clinically, she was tachycardia (104/min), febrile (37.8°C), moderately dehydrated on presentation. Abdominal examination revealed tenderness and rebound tenderness over right iliac fossa with localized guarding. Blood result showed leucocytosis. A preoperative diagnosis of acute appendicitis was made and, an emergency appendicectomy was planned after resuscitation. A grid iron incision was made. A large normal appendix was found with multiple diverticula distributed on both mesenteric and antimesenteric border (Figure 1). Two of them which were adjacent were found inflamed without perforation. Appendicectomy was performed. The patient had an uneventful recovery. The histopathology report confirmed appendicular diverticulitis without any abnormality of the rest of the appendix.

## Discussion

Diverticulum of appendix can rarely be true congenital type and, more commonly false acquired type. The acquired ones are found on the mesenteric border. The incidence of diverticula found in appendicectomy specimens ranges from 0.004% to 0.6% [5]. Patients with appendicular diverticulitis present at an average age of 38 years. It is more common in men and in patients with cystic fibrosis [5,6]. Four subtypes [2], of appendicular diverticulosis have been described in the literature (Table 1). Acute diverticulitis of the appendix has been shown to be more than 4 times as likely as the acute appendicitis to perforate (occurring in 66% of cases), increasing mortality (30-fold compared with acute appendicitis) [2]. Diverticular disease of appendix can be associated with chronic abdominal pain. It can present as an ileo-caecal mass or abscess [7]. In addition, several cases of pseudomyxoma peritonei [8], have been reported from appendicular diverticuli [8].

**Table 1:** Clinical subtypes of appendicular diverticulosis

Type	Diverticulum	Appendix
Type 1	Inflamed	Normal
Type 2	Inflamed	Inflamed
Type 3	Normal	Inflamed
Type 4	Normal	Normal

Appendicular diverticulum can be diagnosed by barium enema. This may make removal of an appendix with diverticulosis appropriate, when found incidentally during surgery or upon barium enema.

## Conclusion

We conclude that inflammatory complications of the appendiceal diverticula, although they may mimic acute appendicitis, are quite distinct clinical entities. Appendicular diverticulosis, when inflamed, carries an earlier and higher rate of perforation. So, even if diagnosed incidentally, appendectomy is justified for appendicular diverticulosis.

## Consent

Written informed consent was obtained from the patient for publication of this case report and accompanying images. A copy of the written consent is available for review by the Editor-in-Chief of this journal.

## Competing interests

The authors declare that they have no competing interest.

## Authors' contributions

SKH is the main contributor in writing the manuscript and preparing the final draft for submission. IK contributed towards reviewing literature. SKH took the photograph and prepared the table. Both the authors read and approved the final manuscript.
